# Supplementary material for: Modulator Combination Improves In Vitro the Microrheological Properties of the Airway Surface Liquid of Cystic Fibrosis Airway Epithelia
Source: Int J Mol Sci. 2022 Sep 27;23(19):11396. doi: 10.3390/ijms231911396 (PMC9569604; doi:10.3390/ijms231911396)
Supplement: Supplementary file 1 [file ijms-23-11396-s001.zip › ijms-1815128-supplementary.pdf]

Modulator Combination Improves In Vitro the Microrheological Properties of the Airway Surface Liquid of Cystic Fibrosis Airway Epithelia

Supplementary Materials

Alessandra Ludovico, Oscar Moran, Debora Baroni*

Istituto di biofisica, Consiglio Nazionale delle Ricerche (CNR), Via De Marini 6, 16149, Genova, Italy

Micro-rheology of glycerol

*To whom correspondence should be addressed: debora.baroni@ibf.cnr.it;
Tel.: +39-0106475559; fax: +39-010647550

Micro-rheology of glycerol

To verify the ability of MPT to provide the parameters characterizing mucus viscosity, we first prepared aqueous solutions containing glycerol at 5 different concentrations: 0%, 20%, 40%, 60% and 80% (V/V), respectively. We added 1 µl of a solution containing the fluorescent beads at 25 µl to the different glycerol samples, and recorded the time course of the position of the beads for 45 seconds for at least fivefold. Images were captured at 1280×960 pixels, at a rate of 5 frames/s. The temperature of the samples was maintained at 22 °C. Figures S1A and S1B show the plots of the squared displacement, <msd>, versus time interval of ~100 beads in 40% and 80% glycerol. The displacement of the beads showed a clear dependence on the glycerol concentration (Figure S1C); figure S1D shows the plot of the apparent diffusion coefficient, D_0, calculated from the <msd> of the beads, versus glycerol concentration. As expected, the values of D_0 depended on the glycerol concentration and this dependence could be fitted with equation (2). Table S1 reports the values of the diffusion coefficient, D_0, the elastic modulus, α, and the viscosity, η, of the different glycerol solutions used to calibrate our MPT system.

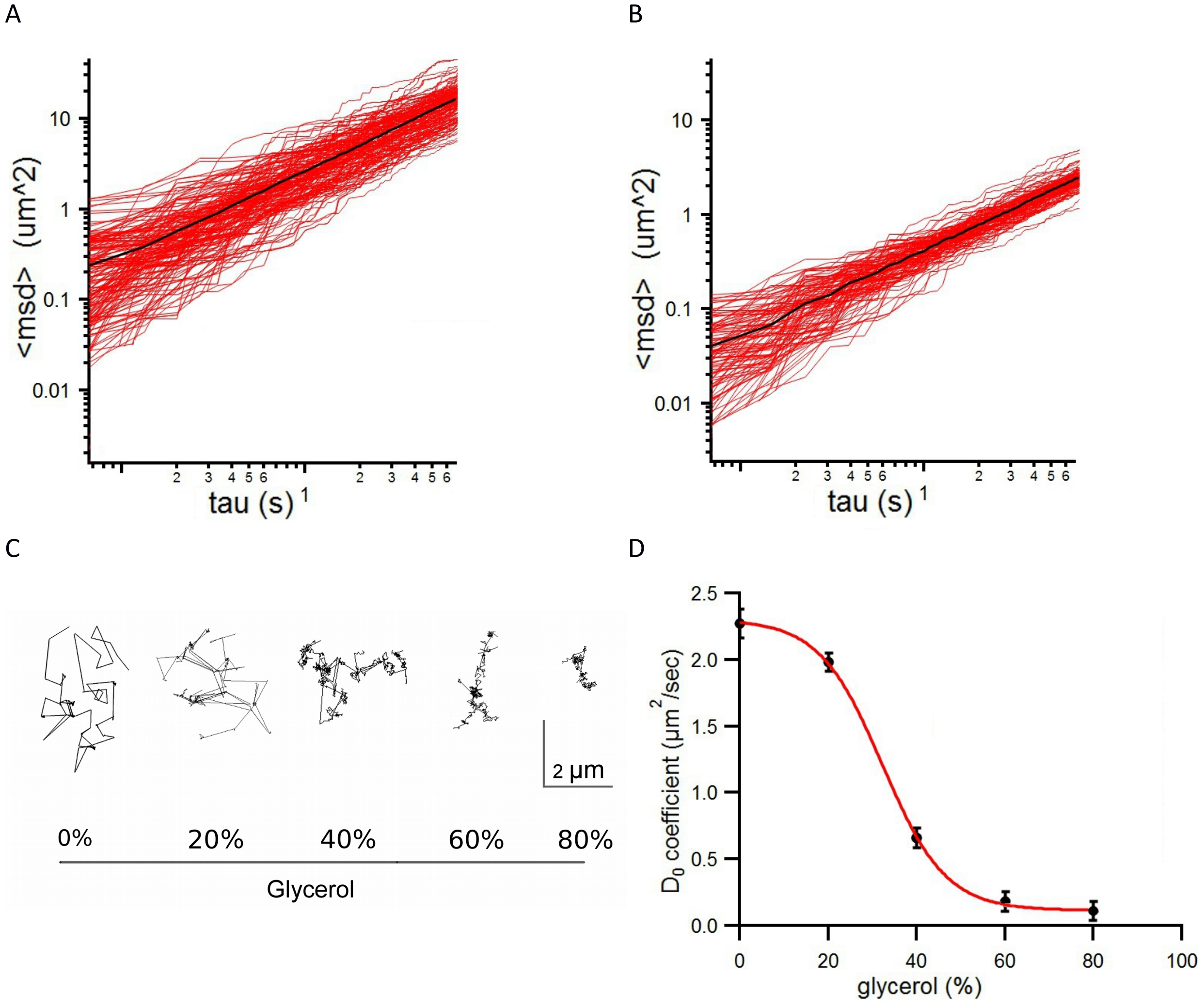

Figure S1.(**A, B**) Plot of the squared displacement versus time interval of ~100 beads in 40% and 80% glycerol. The mean square displacement, <msd>, is shown as a black solid line. (**C**) Displacement of fluorescent beads in solutions containing 0%, 20%, 40%, 40%, 60% and 80% (V/V) glycerol, respectively. (**D**) Dependence of D_0 on glycerol concentration in aqueous solutions.

Table S1. Values of the diffusion coefficient, D_0, the elastic modulus, α, and of the viscosity, η, obtained from different glycerol solutions. Data are expressed as mean ± sem

**Glycerol %(V/V)**	**D_0 (μm^2/sec)**	**α**	**η (cPoise)**
0	2.274 ± 0.011	0.978 ± 0.003	0.950 ± 0.004
20	1.098 ± 0.007	0.957 ± 0.003	1.968 ± 0.012
40	0.660 ± 0.001	0.964 ± 0.001	3.272 ± 0.005
60	0.220 ± 0.001	0.996 ± 0.002	9.833 ± 0.034
80	0.066 ± 0.002	0.988 ± 0.002	32.981 ± 0.134
